# Anodal tDCS over Primary Motor Cortex Provides No Advantage to Learning Motor Sequences via Observation

**DOI:** 10.1155/2018/1237962

**Published:** 2018-03-29

**Authors:** Dace Apšvalka, Richard Ramsey, Emily S. Cross

**Affiliations:** ^1^Social Brain in Action Laboratory, Wales Institute for Cognitive Neuroscience, School of Psychology, Bangor University, Wales, UK; ^2^MRC Cognition and Brain Sciences Unit, University of Cambridge, Cambridge, UK; ^3^Institute of Neuroscience and Psychology, School of Psychology, University of Glasgow, Glasgow, UK

## Abstract

When learning a new motor skill, we benefit from watching others. It has been suggested that observation of others' actions can build a motor representation in the observer, and as such, physical and observational learning might share a similar neural basis. If physical and observational learning share a similar neural basis, then motor cortex stimulation during observational practice should similarly enhance learning by observation as it does through physical practice. Here, we used transcranial direct-current stimulation (tDCS) to address whether anodal stimulation to M1 during observational training facilitates skill acquisition. Participants learned keypress sequences across four consecutive days of observational practice while receiving active or sham stimulation over M1. The results demonstrated that active stimulation provided no advantage to skill learning over sham stimulation. Further, Bayesian analyses revealed evidence in favour of the null hypothesis across our dependent measures. Our findings therefore provide no support for the hypothesis that excitatory M1 stimulation can enhance observational learning in a similar manner to physical learning. More generally, the results add to a growing literature that suggests that the effects of tDCS tend to be small, inconsistent, and hard to replicate. Future tDCS research should consider these factors when designing experimental procedures.

## 1. Introduction

Learning new motor skills is crucial for successful interactions with one's environment. However, the neural mechanisms that underlie skill learning in the human brain are not well known. Most prior neuroscience research has investigated skill acquisition through physical practice. For example, prior studies have shown that motor skill learning can be facilitated by applying anodal transcranial direct-current stimulation (tDCS) to the primary motor cortex (M1) during physical practice of new skills (for reviews, see [1–4]). These results suggest that M1 plays a functional role when learning novel motor skills through physical practice. However, motor learning also occurs when watching others perform actions in the absence of physical practice [5]. To date, the extent to which the motor system operates similarly in physical and observational learning remains unclear. In the present study, therefore, we use anodal tDCS over M1 to determine the extent to which stimulation of the motor system may also facilitate learning via observation.

Motor learning increases excitability of M1 and strengthens synaptic connections within M1 through long-term potentiation- (LTP-) like mechanisms [6–8]. Similarly, applying an anodal current over M1 via tDCS increases excitability of cortical neurons under the surface area of the electrode [9, 10] and the aftereffects of stimulation are believed to be related to LTP-like changes in synaptic plasticity [11]. In addition, combining anodal tDCS over M1 with a motor learning task (so-called “online” stimulation) has been shown to facilitate motor learning [1–4], which suggests that there may be additive effects of combining stimulation techniques with learning paradigms.

Physical practice of motor movements is not essential to learn new skills; motor skills can also be learned by watching others perform actions [5]. Although many studies have shown that motor skills can be learned via observation, the specific neural mechanisms that are required to translate visual input into motor programs are not well understood [12, 13]. Several theories suggest that action observation engages an observer's own motor system by establishing internal representations of the motor programs required to perform the action (for a review, see [14]). Engagement of premotor and parietal cortices is consistently reported during both action execution and action observation, and these two brain regions form the core of the so-called human mirror system [15, 16].

Although M1 is not part of the premotor-parietal mirror system, accumulating evidence suggests that it plays an important role in action observation, as well as learning by observation. Electrophysiological recordings in monkeys have shown that cells in M1 exhibit mirror-like properties, meaning that they respond to both observed and executed movements [17–19]. In humans, repetitive transcranial magnetic stimulation (TMS) over M1, which temporarily disrupts function, effectively inducing a short-lived “virtual lesion,” reduces the benefits of motor learning by observation [20]. Further, M1 engagement during observation might be a critical determinant for the success of motor learning via observation [21]. If M1 plays a similar functional role in observational learning as it does in physical learning, increasing M1 excitability during observational learning should facilitate skill acquisition in a similar manner as that reported for learning by physical practice.

Here, we investigate whether applying anodal tDCS over M1 during observational practice facilitates acquisition and retention of a keypress sequence learning task. We hypothesise that observational practice coupled with anodal tDCS should have beneficial effects on learning compared to observational practice alone, as has been previously reported for learning by physical practice [1–4]. Such a pattern of findings would support the view that M1 plays a similar functional role in learning via observation and physical practice, thus further illuminating the functional mechanisms supporting action and perception links in motor learning.

## 2. Method

### 2.1. Participants

Fifty-five participants consented to participate in the study. Five participants did not finish all sessions, including the posttraining testing sessions. These five participants were thus excluded from analyses as they did not have posttraining performance measures that were critical for testing our hypothesis. The final sample comprised 50 participants: 14 males and 36 females, 18 to 30 years old (*M* = 20.60 years, SD = 2.40). All participants were right-handed (based on self-report) Bangor University student volunteers with normal or corrected-to-normal vision and no history of neurological or psychiatric disorders. Participants reported no contraindications to TMS or tDCS (personal/family history of epilepsy or seizures, metal or implants in the body, frequent headaches, history of serious head injury, heart disease, and possibility of being pregnant) and were not taking any medication that affects brain function (e.g., antiepileptic medication, tranquilizers, or antidepressants). Prior to the first stimulation session, participants were assigned to the sham (*N* = 24) or active stimulation (*N* = 26) group (see Section 2.4 for assignment procedure). No significant differences existed between the groups in terms of demographics and baseline performance (summarised in Table 1). Participants provided their written informed consent prior to beginning all experimental procedures and either received eight course credits or were paid £30 for their participation following completion. The study was conducted in accordance with the Declaration of Helsinki and all procedures were approved by the Ethics Committee of the School of Psychology at Bangor University (protocol 2016-15675) and the UK Ministry of Defence Research Ethics Committee (protocol 735/MODREC/15).

### 2.2. Stimuli

A keypress sequence learning paradigm was implemented, based on the task used by Wiestler and Diedrichsen [22]. A standard QWERTY black computer keyboard had the Q, 3, 4, 5, and Y keys covered with red tape and all surrounding keys removed. In pre- and posttraining sessions, participants were required to press the red keys with the five fingers of their left hand in a specified order. During the observational training tDCS sessions, participants watched videos of the experimenter performing the keypress task. For the video recordings, a similar keyboard was used with the only difference that the sides of the five keys were covered in yellow to improve the visibility of the key being pressed. Stimuli presentation and response recordings were performed using MATLAB 8.3.0 (The MathWorks, MA, USA) and Psychophysics Toolbox 3.0.12 [23].

### 2.2.1. Keypress Sequences

The same set of 12 five-element keypress sequences was used previously by Wiestler and Diedrichsen [22]. Each sequence required the five fingers of the left hand to be pressed once in a sequential order, with each of the 12 sequences featuring a different order with no more than three adjacent finger presses in a row. All sequences were matched for difficulty, based on a previous work [22]. For each participant, from the set of 12 sequences, four sequences were randomly allocated to the trained condition, and four other sequences were allocated to the untrained condition. The remaining four sequences remained unused.

### 2.2.2. Videos

**Figure 1:**
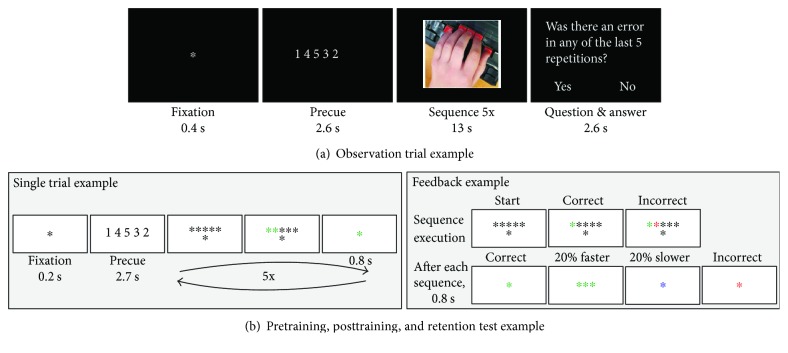
Sequence learning and testing elements. (a) Observation trial example. A sequence cue was followed by a video showing a hand executing the sequence five times, either correctly or incorrectly. Occasionally, a question was asked whether there was an error in any of the five repetitions, and a response had to be made. (b) Execution trial example. A cued sequence had to be memorised and then executed five times while receiving performance feedback.

For the observational training sessions, 13-second videos were created showing the experimenter's left hand from a first-person perspective, slightly tilted to the right (see Figure 1(a) and Supplementary 1). Each video showed the experimenter executing one sequence five times, with naturally varying breaks between each sequence repetition to ensure a more authentic presentation of the performance. For the same reason, for each sequence, five different video versions were recorded. This ensured closer to natural performance variation of the same sequence. An additional video version for each sequence was created where one of the five sequence executions was incorrect. This resulted in 72 videos in total.

Sequences were executed at an intermediate performance level, which was determined by behavioural pilot test results, where the average time to complete a correct sequence execution was 2.29 seconds (pilot: *N* = 17, *M* = 2.29 s, and SE = 0.14). Each original video, showing five repetitions of the same sequence, was slightly sped up or slowed down (±10%) to make it exactly 13 seconds long. Consequently, the authenticity of movement performance was somewhat reduced, but the relative variability within the video remained intact. The average length of time for a single sequence execution in the videos was 2.3 seconds. The videos were presented on a computer monitor in full colour on a black background. The frame rate was 29 frames per second with the resolution of 600 × 526 pixels, showing approximately natural hand size.

### 2.3. Procedure

**Figure 2:**
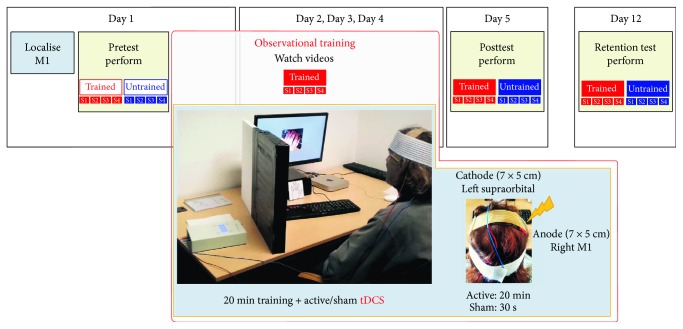
Experimental procedure. The experiment involved pretest, four 20-minute-long training sessions coupled with tDCS, posttest, and retention test. In the pre-, post-, and retention tests, participants executed eight keypress sequences (four of them to be trained, the other four untrained) with the left (nondominant) hand. In the training sessions, participants watched videos of a model's left hand executing four of the eight sequences. During training, participants received either sham or active (1 mA) 20-minute stimulation over the right motor cortex (35 cm^2^ large area centred on the left-hand motor area M1).

Participants were required to watch and learn four different 5-element keypress sequences performed by a model with the left (nondominant) hand. Participants underwent six testing sessions (Figure 2). Consecutive multiple-day stimulation sessions were administered because they generally produce higher tDCS effects compared to single stimulation sessions [1], showing a cumulative increase in cortical excitability [24] and improved motor skill consolidation and retention [25, 26]. On the first day of testing (day 1), participants' left-hand motor area was localised with TMS (see below for details). After the localisation procedure, participants received task instructions and completed three single-sequence execution trials to ensure they understand the task. The familiarisation procedure was followed by a pretest, which was followed immediately by the first observational practice session. The observational practice sessions continued for the next three consecutive days (day 2 to day 4). For most participants, sessions were arranged at the same time of the day as the first practice session (with 1.5 to 2.5-hour difference for three participants in the sham group and 0.5 to 1.5-hour difference for four participants in the active stimulation group). The day after completing the final observational practice session, participants performed a posttest to assess learning (day 5). One week later, they returned to the lab one final time to perform a retention test to assess memory for the different sequences (day 12).

Stimuli presentation and response recordings were performed using MATLAB 8.3.0 (The MathWorks, MA, USA) and Psychophysics Toolbox 3.0.12 [23]). All scripts are available at Github (https://github.com/dcdace/2017_tDCS).

### 2.3.1. Testing Sessions

In the pre-, post-, and retention performance sessions, participants performed four trained and four untrained sequence execution trials in a random order with the left hand. Each trial consisted of five repetitions of the same sequence. All trial-related information was presented centrally at the bottom of the screen against a grey background. A trial started with a black fixation cross (0.2 s), followed by the sequence cue presented as five digits (2.7 s) that indicated from right to left which key to press: “1”—the right-most key pressed with the thumb and “5”—the left-most key pressed with the little finger (see Figure 1(b)). After the cue, the digits were replaced by the fixation cross and five black asterisks above it. This served as a “go” signal to execute the memorised sequence five times as quickly and accurately as possible. If the correct key was pressed, the corresponding asterisk on the screen turned green, if a wrong key was pressed, the asterisk turned red.

After executing a single sequence, the central fixation cross changed colour to provide feedback on the performance (0.8 s): green—correct sequence execution, red—incorrect sequence execution, blue—correct, but executed 20% slower than the median execution time (ET) in the previous trials, and three green asterisks—correct and executed 20% faster than the median ET in the previous trials. After this short feedback, all asterisks turned black signalling the start of the next execution trial. After five executions of the same sequence, the trial ended and the next sequence was cued.

Participants' performance was assessed as the average sequence initiation time, execution time, and error rate for the four trained (to-be-trained) and the four untrained sequences. The error rate was measured as the percentage of incorrect sequence executions. Incorrectly executed trials were excluded from initiation time and execution time measurements. The initiation time was measured as the duration between the “go” signal and the first keypress. The execution time was measured as the duration between the first and fifth keypresses.

### 2.3.2. Observational Training Sessions

During the observational training sessions, participants received either sham or active brain stimulation while watching videos of the model's left hand executing four sequences. Each video showed five repetitions of the same sequence. A trial started with a 5-digit cue (for 2.6 s), indicating the sequence to be executed, followed by a video (13 s) showing five executions of the cued sequence. Participants were instructed to watch whether the hand executed the correct sequence all five times. Occasionally, participants were asked whether there was an error in any of the five executions—the error question.

Each practice session was divided into three blocks, separated by a one-minute rest period. Within each block, 20 videos were presented in a random order: each sequence video four times and one “error video” (with at least one incorrect sequence execution) for each sequence. The error question was asked randomly 5–7 times per block. At the end of each block, participants received feedback on how accurately they spotted the incorrect sequence executions. During each session, participants saw a correct execution of each sequence at least 60 times (3 blocks, 4 videos per block, 5 repetitions per video, plus some correct repetitions in the “error video”). The whole training session lasted approximately 20 minutes and was coupled with 20 minutes of sham or active tDCS.

### 2.4. Motor Cortex Stimulation

#### 2.4.1. Right M1 Localisation

Single-pulse TMS was used to localise the left-hand motor area. The TMS coil was positioned on the right hemisphere, slightly anterior and ventral to the vertex of the skull to induce a muscle twitch in the relaxed fingers of the left hand. The stimulator output was started at 45% and increased in steps of 2–5% until a visible twitch was observed. The stimulator output never exceeded 80% and participants received no more than 20 total pulses in total, with an interpulse interval kept to at least 5 seconds. The optimal location at which TMS evoked a just-noticeable finger twitch was marked on the participant's scalp with a surgical marker. For nine participants, a visible twitch was not observed following this procedure and the motor hand area was instead marked per position C4 of the EEG 10-20 system (after [27]). The localisation procedure was performed only on the first testing session and the marked M1 location was renewed with the surgical marker before each stimulation session.

The nine participants whose M1 area could not be localised using TMS were assigned to the sham group as the precise location of the stimulated area was not critical for sham stimulation. We acknowledge that random assignment, independent of localisation procedure, would have been a better approach. The reasons why we could not evoke a visible twitch in some participants may include extent of representation of the hand area and/or its accessibility via the cortical surface. To ensure that any group differences are not driven by the nonrandom assignment to groups, we repeated the main analyses of observational training and stimulation effects with the nine non-TMS-localised participants excluded. The results of this analysis (see Supplementary Materials 1) suggest that nonrandom group assignment did not systematically bias our findings.

### 2.4.2. Stimulation Parameters

We performed a single-blinded protocol. Participants were semirandomly assigned to the sham or active stimulation group, keeping gender balanced between the groups and ensuring that the motor hand area of the active group was localised using the TMS procedure described above. Participants were told that they would receive stimulation for up to 20 minutes, not specifying the exact length of the stimulation and not revealing the existence of two stimulation groups. During each practice session, the sham group received 30 seconds and the active group received 20 minutes of tDCS (cf. [28]).

A 1 mA constant current was delivered using a battery-driven DC-Stimulator Plus (neuroConn GmbH, Ilmenau, Germany) via a pair of conductive rubber electrodes placed into saline-soaked sponges (7 × 5 cm; 0.029 mA/cm^2^ current density). The electrodes were secured with elastic bands. The contact impedance was monitored throughout the session to ensure it stays below 15 k*Ω*.

The anode was centred over the previously marked right M1. Due to the electrode size, the stimulation likely extended into premotor and anterior parietal cortices as well. The cathode was placed on the left supraorbital ridge (see photographs in Figure 2). The current was ramped up to 1 mA over 10 seconds, held constant for either 30 seconds (sham) or 20 minutes (active), and then ramped down over 10 seconds. This method is recommended to reliably blind participants to stimulation condition and ensure similar sensations for sham and active stimulation groups [28].

The observational training task started one minute after stimulation onset, to allow time for participants to adapt to the stimulation sensations and to ensure they felt comfortable with carrying on with the task. The stimulation ended about one minute before the end of the task.

### 2.4.3. Sensation Questionnaire

After each training session, participants provided information on the intensity of experienced sensations (itching, pain, burning, heat, pinching, metallic taste, and fatigue), the timing of any discomfort (when did the discomfort begin and how long did it last?), and the perceived impact of the stimulation on their performance (adapted from [29]). At the end of the experiment (day 12), participants were debriefed and asked whether they think they received sham or active stimulation.

### 2.5. Data Analysis

All statistical analysis was performed using R (v3.3.2, 2016-10-31) in RStudio (v1.0.136, 2016-12-21, RStudio Inc., Boston, MA). Graphs were produced in MS Excel 2016 (Microsoft, Redmond, WA, USA). The Excel files, raw data, and scripts with all analysis procedures and for reproducing results are available at https://github.com/dcdace/2017_tDCS.

Given the total sample size of 50, the study had 80% power to detect effects of tDCS that are conventionally considered large (Cohen's *d* = 0.71; the effect size was estimated with a *power.t.test* function in *R* for a two-sample, one-sided *t*-test with 25 observations per group). Three previous multiple stimulation session (3–5 consecutive days, 20–25 min per day, 1-2 mA, and ~12.5 participants per group) M1 anodal-tDCS physical training studies reported large tDCS effects ranging from 0.95 to 1.33 Cohen's *d* [25, 26, 30].

**Figure 3:**
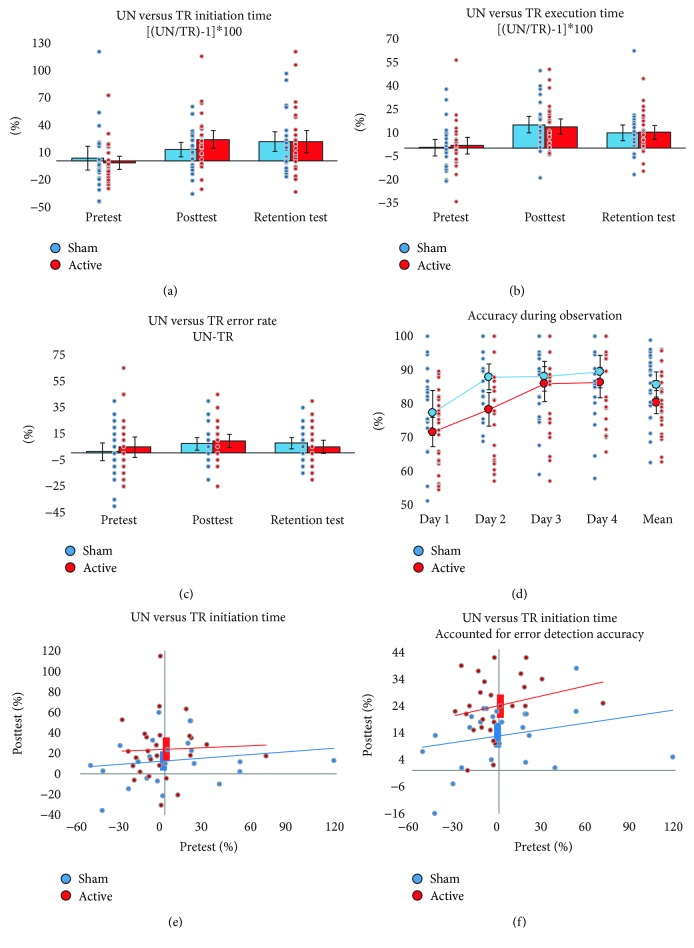
Performance results. Pre-, post-, and retention test differences in initiation time (a), execution time (b), and error rate (c) between trained (TR) and untrained (UN) sequences for sham (blue) and active (red) stimulation groups. (d) Error detection accuracy during observational practice sessions. (a–d) Bars and large dots: group averages; small dots: individual participant values; error bars: 95% CI (one-tailed for (a), (b), and (c); two-tailed for (d)). (e) Regression lines of pretest (predictor) and the posttest differences between trained and untrained sequence initiation times for the sham (blue) and active (red) stimulation groups. Intercepts of the regression lines represent the predicted posttest difference if the pretest difference is zero. Vertical bars represent 95% CIs (one-tailed) of intercepts (f). Same as (e), but posttest difference corrected for error detection accuracy during training sessions.

The effect of observational training on sequence-specific learning was assessed as a posttraining difference between the trained and untrained sequence initiation time, execution time, and error rate. For the sequence initiation time and execution time, we measured a percentage difference ([(untrained/trained) − 1]^∗^100), but for the error rate (to avoid dividing by zero), we calculated an absolute difference (untrained-trained) between the trained and untrained sequences. Results for all of these measures are plotted in Figures 3(a)–3(c) (raw performance measures are provided in Supplementary Materials 2). To correct for possible pretraining differences, we performed a linear regression between the pretraining difference (predictor) and the posttraining difference (outcome; see Figure 3(e) for an example plot). The intercept of the regression line was used as a measure of the posttraining difference between trained and untrained sequences, controlling for possible pretraining differences. This method reduces the noise of unwanted differences in the difficulty of trained and untrained sequences and thus allows a more accurate measurement of the training effect.

For the assessment of tDCS effects, we complemented null hypothesis significance testing with a Bayesian analysis to provide evidence for the null result. We used the *generalTestBF* function of the R package BayesFactor v0.9.12-2 [31] with its default parameters. The Bayesian test produced a Bayes factor to allow quantification of evidence in favour of either the alternative (BF_10_) or null (BF_01_) hypothesis based on prior beliefs and the present data. To describe the Bayes factor results, we used Jeffreys' [32] classification scheme and reported both BF_10_ and BF_01_. Jeffreys proposed benchmarks for evaluating the strength of evidence as anecdotal (BF_10_ 0–3), substantial (BF_10_ 3–10), and strong (BF_10_ 10–30). These Bayes Factors can be readily interpreted as a ratio of evidence in favour of the experimental effect compared to the null effect. For example, a BF_10_ of 3 would represent that the experimental effect is three times more likely than the null, given the data.

The significance threshold for all statistical comparisons was *p* < 0.05. If not specified otherwise, all sample means are reported with their 95% confidence intervals in square brackets. Confidence intervals for two-tailed tests were calculated as SE ^∗^ 2.07 for the sham group (df 23) and SE ^∗^ 2.06 for the active group (df 25), whereas confidence intervals for one-sided tests were calculated as SE ^∗^ 1.71 for df 23 and df 25 [33].

## 3. Results

### 3.1. Group Characteristics and Sensations during Training Sessions

**Figure 4:**
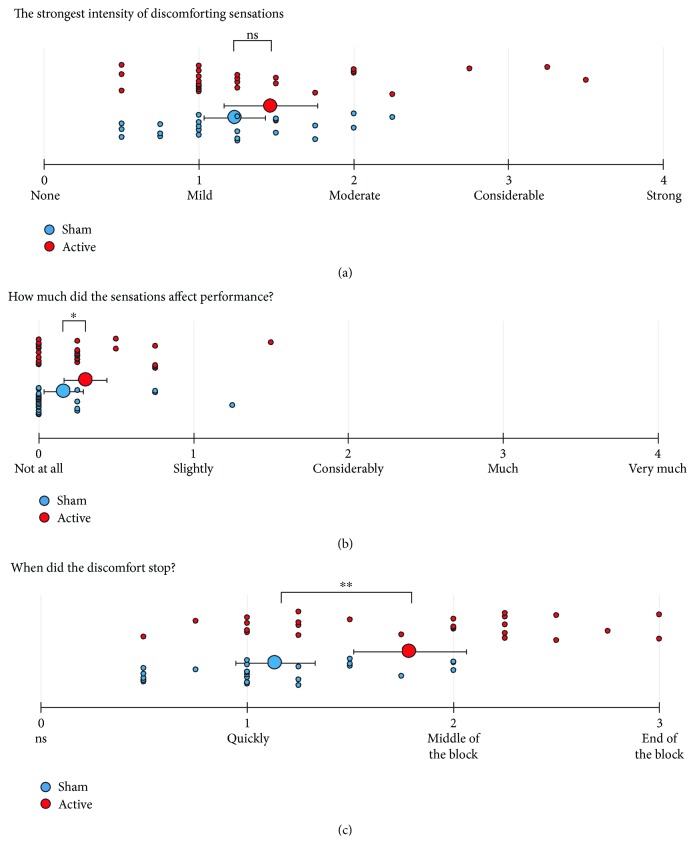
The 4-day average values of self-reported sensations during the training sessions. Large dots: group averages; small dots: individual participant values; red: active; blue: sham; error bars: 95% CI, two-tailed; ^∗^ *p* < 0.05, ^∗∗^ *p* < 0.01, two-tailed.

Gender proportion between the sham and active stimulation groups was compared using a chi-square test. Mann–Whitney *U* tests were used to compare group age and experienced sensations during the training sessions. Participants' baseline performance (pretraining average of trained and untrained sequences) was compared using a two-tailed independent-measures *t*-test. Results are summarised in Table 1. The reported sensations for each training day are summarised in Table 2 and averages of all training days are plotted in Figure 4.

There were no differences in gender, age, and baseline performance between the groups. On average, both groups reported mild to moderate levels of discomfort during stimulation with no significant difference between the groups (Table 1; Figure 4(a)). Although the active stimulation group did report a small but significantly larger impact of stimulation on performance than the sham group, the perceived impact for both groups was closest to zero (“no impact”) (Table 1; Figure 4(b)). Finally, sensations lasted significantly longer for the active compared to the sham group (Figure 4(c)), with average sensations stopping between “quickly” and “in the middle of the block” across both groups.

The reported sensation data, therefore, shows that there were small but significant sensation differences between the sham and active stimulation groups. The sham protocol should provide comparable sensations to the active stimulation protocol [28]. However, small but significant sensation differences between the stimulation groups, using comparable protocols to ours, have been reported before [29], raising an issue that the widely accepted sham stimulation procedure may not be sufficiently effective.

Following the recommendation of Fertonani et al. [29], at the end of the experiment, we asked participants whether they think they received sham or active stimulation. In total, 54% thought they received active stimulation, 32% thought they received sham stimulation, and 14% did not know. There was no significant difference between the two groups in terms of which kind of stimulation they thought they received (*χ* ^2^ = 1.24, *p* = 0.538), thus confirming the success of the blinding procedure.

### 3.2. Accuracy during Training Sessions

During the observational practice sessions, attention to the task was assessed by accurate responses to the error question (spotting incorrectly executed sequences). The overall accuracy was 83%, significantly higher than a 50% chance level (yes/no answers; *t*_49_ = 24.61, *p* < 0.001, two-tailed), confirming that participants paid attention to the task. The average accuracies for each group and day are plotted in Figure 3(d). On average, across the four training days, the sham group performed better (*M* = 86% (82%, 90%)) than the active group (*M* = 81% (77%, 85%)), with a marginally significant difference between the two groups (Welch two-sample *t*-test for nonequal variance: *t*_47.27_ = 1.99, *p* = 0.052, two-tailed, *d* = 0.56).

The small difference in error detection accuracy between the groups was an unexpected finding. It cannot be ruled out that anodal tDCS of M1 had some negative effects on the error detection accuracy. However, we do not have any a priori or theoretical grounds to support this suggestion. Another possibility is that the error detection accuracy was influenced by the discomforting sensations during the training sessions that, as reported above, affected the stimulation group more than the sham group. This possibility is supported by a significant negative correlation between the average error detection accuracy and the average self-report on how much performance was affected by the discomforting sensations (Kendall's tau-*b* = −0.296, *p* = 0.008, two-tailed; across both groups).

The lower error detection accuracy for the active stimulation group raises a possibility that the active group may not have been able to learn from the videos as well as the sham group due to stimulation-related discomfort and consequent impact on attention. To account for this possibility, we complement the planned analysis with an exploratory analysis that includes mean error detection accuracy as a covariate when assessing the stimulation effect.

### 3.3. Observational Training Effects on Sequence-Specific Learning

Both groups showed significant observational training effects at both posttest and retention test on all three performance measures, with medium to large effect sizes for the performance difference between trained and untrained sequences (*d*_z_ = 0.52–1.02; comparable to previous reports on keypress sequence learning by observation, e.g., [34–36]). The only exception to this pattern of results was that the active stimulation group demonstrated no effect on error rates at the retention test. Detailed results are provided in Table 3, columns I and II, where *B*_0_ represents the percentage performance improvement from pretest. All tests in Table 3 are one-tailed as we were testing a directional prediction for the difference between trained and untrained sequences. Furthermore, Supplementary Materials 3 document the extent to which the training manipulation generalised to the untrained sequences, comparing the active and sham stimulation groups.

### 3.4. tDCS Effects on Sequence-Specific Learning by Observation

#### 3.4.1. Primary Analysis

The effect of stimulation on sequence-specific learning was assessed by comparing observational training effects (the posttraining~pretraining regression line intercepts) between the sham and active stimulation groups. The performed analysis of covariance (ANCOVA) did not reveal any significant difference between the two groups on any of the three measures either at posttest or retention test (Figure 3(e) plots posttest initiation time results; see Supplementary Materials 4 for ANCOVA results of the raw means). The Bayes factor analyses yielded anecdotal to substantial evidence against the stimulation effect. Detailed results are provided in Table 3, column III (reporting significance of the group as a predictor variable for the training effect).

#### 3.4.2. Secondary Analysis: Accounting for Error Detection Accuracy

Due to error detection differences between the groups, in an exploratory analysis, we added mean error detection accuracy as a covariate to the previous ANCOVA model and repeated the group comparison analysis. This exploratory analysis revealed evidence for the stimulation effect on the percentage difference between trained and untrained sequence initiation times at posttest. Compared to the sham group, the active stimulation group showed greater difference on this measure (see Figure 3(f)). The error detection accuracy significantly predicted the outcome (*β* = 0.431, *p* = 0.003; the better the accuracy during training, the faster initiation time of trained relative to untrained sequences at posttest). All other measures showed substantial to strong evidence against the stimulation effect when accounting for the error detection accuracy. Detailed results are provided in Table 3, column IV (reporting significance of the group as a predictor variable for the training effect accounting for the error detection accuracy).

## 4. Discussion

We investigated the extent to which anodal tDCS over M1 facilitates motor sequence learning by observation, as previously reported for learning by physical practice [1–4]. Both the active and sham stimulation groups benefited from observational practice, replicating previous findings that motor skills can be learned by observation without overt physical practice [5, 34–39]. However, active stimulation over M1 did not provide an advantage to learning the motor sequences through observation over and above sham stimulation. Furthermore, Bayesian analyses revealed anecdotal to substantial evidence in favour of the null hypothesis across our dependent measures. Our findings therefore do not provide strong support for the hypothesis that excitatory M1 stimulation can enhance observational learning in a similar manner to physical learning.

### 4.1. Understanding the Role of the Motor System during Observational Learning

Although there is a consensus that shared mechanisms exist between action observation and execution [14], the role played by the motor system in observational learning is not clear [12, 13]. Indeed, several studies have questioned the notion of motor-driven learning by observation, arguing instead that it is driven by perceptual and cognitive processes [40–42]. It is possible, therefore, that primary motor areas might be engaged during action observation [43–45], but their involvement might not be *critical* in shaping observational learning.

Alternatively, it is possible that the effect of anodal tDCS over M1 during observational learning is smaller than during physical learning and subtler than we could detect in the current study. The current study had 80% power to detect an effect size that is typically considered large (0.71 Cohen's *d*). Therefore, we have reasonable confidence that we could detect large effects of stimulation, similar to what were reported previously during physical learning, should they exist. In addition, we followed recommended stimulation protocols by stimulating on consecutive days to enhance effects of stimulation [1] and skill learning [25, 26] (although, see work by Monte-Silva and colleagues [46] that demonstrates the abolishment of LTP-like plasticity in motor cortex when follow-up stimulation occurs 24 hours after initial stimulation). As such, we designed the experiment to increase the likely impact of tDCS on skill learning, but nonetheless report a null result. We suggest that future studies wishing to further explore the role of M1 in observational learning use a similar protocol, but with larger sample sizes, in order to increase statistical power to detect smaller effects.

The null result we report here adds to a growing set of null results using tDCS in tasks ranging from working memory [47, 48] to language [49, 50]. In addition, several recent meta-analyses document conflicting evidence regarding the efficacy of tDCS in a variety of paradigms where effects have previously been reported, as well as growing scepticism regarding a causal role of tDCS in performance enhancement [48, 50]. Given concerns over publication bias in general [51] and in the domain of tDCS in particular [52], it is important to report null results in order to provide a less biased estimate of the likely effect sizes that tDCS may have on behaviour. Therefore, balanced reporting of null results (in addition to positive results, such as those observed with tDCS over premotor cortex facilitating observational learning of a motor sequence [53]) will help to build a cumulative science of observational learning and tDCS. For instance, based on the details of the current study, researchers who wish to further explore the relationship between primary motor cortex activity and observational learning will have a more accurate estimate of the likely effect sizes that they might be targeting, which will directly inform power calculations and study design decisions.

The current study also provides a platform for future tDCS studies to build upon in other ways. Indeed, there are many avenues that future work could pursue in order to probe the relationship between the motor system and observational learning. For example, the effects of tDCS on observational learning may be task dependent. Aridan and Mukamel [21] reported a positive relationship between M1 activity during action observation and the success of motor skill learning via observation only if the observed model's performance was faster than the observer's performance at baseline. The current study used an intermediate model, which may not have been challenging enough to engage the motor system sufficiently. Future studies could use an expert model whose performance consistently exceeds the observer's baseline performance to test this possibility directly.

Follow-up work could also investigate the impact of different stimulation protocols. For example, several reports demonstrate a powerful effect of dual-M1 stimulation on motor learning [30, 54], which outperforms unilateral M1 stimulation montages [55–58]. Another possibility to explore with future work is the impact of tDCS intensity on motor learning effects. Recent work demonstrates that 1.5 mA, but not 1.0 mA, anodal tDCS over M1 reliably facilitates motor learning [59], which raises the possibility that our stimulation intensity was not optimised to induce reliable results. A further consideration is that small differences were observed in the sensations associated with active compared to sham stimulation, which is consistent with prior research [29]. The impact that such sensation differences have on task performance are worth studying in order to more effectively design sham protocols. Moreover, due to the electrode size (7 × 5 cm), the focality of tDCS stimulation is necessarily imprecise, and stimulation in our study may have extended beyond M1 into nearby premotor and anterior parietal brain regions as well. The modulation of cortical excitability under and between the electrodes is still under debate and investigation [10, 60]. As these suggestions demonstrate, many different lines of inquiry will be needed to better understand the relationship between motor system engagement and observational learning.

## 5. Conclusions

Our results do not support the hypothesis that anodal tDCS over M1 facilitates skill learning through observation to a large degree. The null finding does not necessarily imply that the motor system is not involved in sequence learning by observation. Rather, the results suggest that using the parameters employed in the current study, anodal tDCS over M1 does not reliably enhance observational learning. Given that no prior study has used tDCS over M1 in an attempt to enhance observational learning, this finding makes an important contribution to the literature by informing future brain stimulation studies and offering a platform upon which to base further investigation into the role of primary motor cortex in observational learning.

## Figures and Tables

**Table 1:** Group characteristics and self-reported sensations during training sessions.

	Sham (*N* = 24)	Active (*N* = 26)	Group difference (*p* value, effect size)
Demographics			
Gender (male/female)	8 : 16	6 : 20	0.623
Age (years; *M* ± SD)	20.96 ± 2.97	20.27 ± 1.71	0.446, *d* = 0.217
Baseline performance			
Pretest initiation time (s; *M* ± SD)	0.77 ± 0.25	0.89 ± .30	0.117, *d* = 0.455
Pretest execution time (s; *M* ± SD)	1.92 ± 0.57	2.02 ± 0.68	0.590, *d* = 0.153
Pretest error rate (%; *M* ± SD)	25 ± 13	30 ± 15	0.203, *d* = 0.366
Sensations			
Strongest (*M* ± SD)	1.23 ± 0.49	1.46 ± 0.79	0.478, *d* = 0.202
*Affected (M ± SD)*	*0.16 ± 0.32*	*0.30 ± 0.36*	*0.037, d = 0.618*
*Lasted (M ± SD)*	*1.14 ± 0.48*	*1.79 ± 0.71*	*0.001, d = 1.04*

Items in italics (last two rows) highlight variables that significantly differed between the sham and active stimulation groups. Strongest: the strongest reported sensation intensity level (0–4); affected: how much did sensations affect performance (0–4); lasted: when did the discomfort stop (0–3).

**Table:** (a) The strongest intensity of discomforting sensations

Intensity level	Day 1						Day 2						Day 3						Day 4				
	0	1	2	3	4		0	1	2	3	4		0	1	2	3	4		0	1	2	3	4
Sham	1	12	10	1	—		4	11	8	1	—		2	15	7	—	—		5	14	5	—	—
Active	2	11	8	3	2		2	18	2	3	1		2	15	4	2	3		3	16	5	2	—

0: none; 1: mild; 2: moderate; 3: considerable; 4: strong.

**Table:** (b) How much did the sensations affect performance?

Intensity level	Day 1						Day 2						Day 3						Day 4				
	0	1	2	3	4		0	1	2	3	4		0	1	2	3	4		0	1	2	3	4
Sham	19	5	—	—	—		20	4	—	—	—		22	2	—	—	—		21	2	1	—	—
Active	18	7	—	1	—		20	6	—	—	—		18	7	1	—	—		20	6	—	—	—

0: not at all; 1: slightly; 2: considerably; 3: much; 4: very much.

**Table:** (c) When did the discomfort stop?

Intensity level	Day 1					Day 2					Day 3					Day 4			
	ns	1	2	3		ns	1	2	3		ns	1	2	3		ns	1	2	3
Sham	1	15	4	4		4	14	4	2		2	19	3	—		5	18	—	1
Active	2	6	9	9		2	11	7	6		2	8	7	9		3	9	8	6

ns: no sensations; 1: quickly; 2: middle of the block; 3: end of the block.

**Table 3:** Observational practice effects and tDCS effects on sequence-specific learning.

		I	II	III	IV
		Observational training effect (trained versus untrained performance)		Primary results	Secondary results
				tDCS effect (group difference)	tDCS effect (group difference), accounting for accuracy during training sessions
		Sham	Active		
Initiation time	Post	*t*_(22)_ = 2.65, *p* = 0.008, *B*_0_ = 13%, *d*_z_ = 0.54	*t*_(24)_ = 4.02, *p* < 0.001, *B*_0_ = 24%, *d*_z_ = 0.79	*t_(47)_ = 1.50, p* = 0.072*, d = 0.44, anecdotal evidence against the effect (BF_10_/BF_01_ = 0.70/1.43)*	*t*_(46)_ = 2.48, *p* = 0.008, *d* = 0.73, anecdotal evidence for the effect (BF_10_/BF_01_ = 2.41/0.41)
	Ret.	*t*_(22)_ = 3.21, *p* = 0.002, *B*_0_ = 21%, *d*_z_ = 0.66	*t*_(24)_ = 2.87, *p* = 0.004, *B*_0_ = 21%, *d*_z_ = 0.56	*t_(47)_ = 0.05, p* = 0.480*, d = 0.01, substantial evidence against the effect (BF_10_/BF_01_ = 0.29/3.49)*	*t_(46)_ = 0.01, p* = 0.496*, d = 0, substantial evidence against the effect (BF_10_/BF_01_ = 0.29/3.45)*

Execution time	Post	*t*_(22)_ = 5.02, *p* < 0.001, *B*_0_ = 15%, *d*_z_ = 1.02	*t*_(24)_ = 4.75, *p* < 0.001, *B*_0_ = 14%, *d*_z_ = 0.93	*t_(47)_ = −0.37, p* = 0.355*, d = 0.11, substantial evidence against the effect (BF_10_/BF_01_ = 0.30/3.31)*	*t_(46)_ = −0.49, p* = 0.312*, d = 0.15, substantial evidence against the effect (BF_10_/BF_01_ = 0.31/3.20)*
	Ret.	*t*_(22)_ = 4.02, *p* < 0.001, *B*_0_ = 10%, *d*_z_ = 0.82	*t*_(24)_ = 3.99, *p* < 0.001, *B*_0_ = 10%, *d*_z_ = 0.78	*t_(47)_ = −0.06, p* = 0.475*, d = 0.02, substantial evidence against the effect (BF_10_/BF_01_ = 0.28/3.55)*	*t_(46)_ = −0.02, p* = 0.492*, d = 0.01, substantial evidence against the effect (BF_10_/BF_01_ = 0.29/3.43)*

Error rate	Post	*t*_(22)_ = 2.56, *p* = 0.009, *B*_0_ = 7%, *d*_z_ = 0.52	*t*_(24)_ = 2.89, *p* = 0.004, *B*_0_ = 9%, *d*_z_ = 0.57	*t_(47)_ = 0.47, p* = 0.322*, d = 0.14, substantial evidence against the effect (BF_10_/BF_01_ = 0.31/3.20)*	*t_(46)_ = 0.20, p* = 0.422*, d = 0.06, substantial evidence against the effect (BF_10_/BF_01_ = 0.31/3.28)*
	Ret.	*t*_(22)_ = 2.99, *p* = 0.004, *B*_0_ = 7%, *d*_z_ = 0.61	*t_(24)_ = 1.45, p* = 0.08*, B_0_ = 4%, d_z_ = 0.28*	*t_(47)_ = −0.81, p* = 0.210*, d = 0.24, anecdotal evidence against the effect (BF_10_/BF_01_ = 0.37/2.71)*	*t_(46)_ = −1.05, p* = 0.149*, d = 0.31, anecdotal evidence against the effect (BF_10_/BF_01_ = 0.44/2.27)*

Items in italics highlight nonsignificant effects. All *p* values reported reflect one-tailed tests as we had directional predictions for the influence of training and stimulation on our performance measures. Results are uncorrected for multiple comparisons.

## Supplementary Materials

Supplementary 1 The Supplementary Materials (videos) illustrate one example trial of an observational learning video stimulus.

Supplementary 2 Supplementary Materials 1 contains full results excluding the nine participants for whom TMS localisation of M1 was not possible. These supplementary materials contain a table of the observational practice effects and tDCS effects on sequence-specific learning, as well as a figure visualising all performance results excluding these nine participants. Supplementary Materials 2 is a figure visualising the mean and standard deviation values of participants' initiation time, execution time, and error rate, split into pre, post, and retention tests, and by the stimulation group (active versus sham). Supplementary Materials 3 includes a table documenting group differences in performance generalisation to the untrained sequences. Finally, Supplementary Materials 4 includes the tDCS effects on sequence-specific learning using ANCOVA.
