# Supplementary material for: Anodal tDCS over Primary Motor Cortex Provides No Advantage to Learning Motor Sequences via Observation
Source: Neural Plast. 2018 Mar 29;2018:1237962. doi: 10.1155/2018/1237962 (PMC5896271; doi:10.1155/2018/1237962)
Supplement: Supplementary 2 — Supplementary Materials 1 contains full results excluding the nine participants for whom TMS localisation of M1 was not possible. These supplementary materials contain a table of the observational practice effects and tDCS effects on sequence-specific learning, as well as a figure visualising all performance results excluding these nine participants. Supplementary Materials 2 is a figure visualising the mean and standard deviation values of participants' initiation time, execution time, and error rate, split into pre, post, and retention tests, and by the stimulation group (active versus sham). Supplementary Materials 3 includes a table documenting group differences in performance generalisation to the untrained sequences. Finally, Supplementary Materials 4 includes the tDCS effects on sequence-specific learning using ANCOVA. [file 1237962.f2.docx]

## Supplementary Materials 1: Results without the nine non-TMS localised participants

### Accuracy during training sessions

During the observational practice sessions, attention to the task was assessed by accurate responses to the error question (spotting incorrectly executed sequences). The overall accuracy was 82%, significantly (p < 0.001) higher than a 50% chance level (yes/no answers), confirming that participants paid attention to the task. The average accuracies for each group and day are plotted in Figure 1D. On average, across the four training days, the sham group performed better (M = 85% [79%, 91%]) than the active group (M = 81% [77%, 85%]), with no significant difference between the two groups (t_26.44_ = 1.35, p = 0.189). There was a negative correlation between the average accuracy and the average self-report on how much performance was affected by the discomforting sensations (Kendall’s tau-b = -0.256, p = 0.042; across both groups).

### Observational training effects on sequence-specific learning

The effect of observational training on sequence-specific learning was assessed as a post-training (separately for the post-test and retention-test) difference between the trained and untrained sequence initiation time, execution time, and error rate. For the sequence initiation time and execution time, we measured a percentage difference ([(untrained/trained)-1]*100), but for the error rate (to avoid dividing by zero), we calculated an absolute difference (untrained-trained) between the trained and untrained sequences (results of these measures are plotted in Figure 1A-C). To correct for possible pre-training differences, we performed a linear regression between the pre-training difference (predictor) and the post-training difference (outcome; see Figure 1E for an example plot). The intercept of the regression line was used as a measure of the post-training difference between trained and untrained sequences, controlling for possible pre-training differences. This method reduces the noise of unwanted differences in the difficulty of trained and untrained sequences and thus allows a more accurate measurement of the training effect.

Both groups showed significant observational training effects at both post-test and retention-test on all three performance measures, with medium to large effect sizes (d_z_ = 0.52 – 1.02). Except, the active stimulation group demonstrated no effect on error rates at retention-test. Detailed results are provided in Table 1.

### tDCS effects on sequence-specific learning by observation

#### Primary analysis

The effect of stimulation on sequence-specific learning was assessed by comparing observational training effects (the post-training ~ pre-training regression line intercepts) between the sham and active stimulation groups. The performed analysis of covariance (ANCOVA) did not reveal any significant difference between the two groups on any of the three measures either at post-test or retention-test. (Figure 1E plots post-test initiation time results). The Bayes factor analysis returned anecdotal to substantial evidence against the stimulation effect. Detailed results are provided in Table 1.

#### Secondary analysis: accounting for error detection accuracy

Due to concern that the stimulation effect could be confounded by sensation and error detection differences (both of which were negatively correlated) between the sham and active stimulation groups, we added the mean error detection accuracy as a covariate to the previous ANCOVA model and repeated the group comparison analysis.

The corrected analysis revealed evidence for the stimulation effect on the percentage difference between trained and untrained sequence initiation times at post-test. Compared to the sham group, the active stimulation group showed a greater difference on this measure (see Figure 1F). The error detection accuracy significantly predicted the outcome (β = 0.554, p < 0.001; the better the accuracy during training, the faster initiation time of trained relative to untrained sequences at post-test). All other measures showed anecdotal to substantial evidence against the stimulation effect when accounting for the error detection accuracy. Detailed results are provided in Table 1.

Table 1. Observational practice effects and tDCS effects on sequence-specific learning with nine non-TMS localised participants excluded.

|  | | **Observational training effect**  **(trained vs. untrained performance)** | | **tDCS effect**  **(group difference)** | **tDCS effect,**  **accounted for the accuracy during training sessions** |
| --- | --- | --- | --- | --- | --- |
|  |  | **Sham (N = 15)** | **Active (N = 26)** |  |  |
| **Initiation time** | **Post** | t_(13)_ = 1.95, p = 0.073,  B_0_ = 11%, d_z_ = 0.50. | t_(24)_ = 4.02, p < 0.001,  B_0_ = 24%, d_z_ = 0.79. | t_(38)_ = 1.50, p = 0.141, d = 0.49,  anecdotal evidence against the effect  (BF_10_/BF_01_ = 0.76/1.31). | t_(37)_ = 2.69, p = 0.011, d = 0.89,  substantial evidence for the effect  (BF_10_/BF_01_ = 3.44/0.29). |
|  | **Ret.** | t_(13)_ = 2.67, p = 0.019,  B_0_ = 25%, d_z_ = 0.69. | t_(24)_ = 2.87, p = 0.008,  B_0_ = 21%, d_z_ = 0.56. | t_(38)_ = -0.35, p = 0.729,  substantial evidence against the effect  (BF_10_/BF_01_ = 0.33/3.00). | t_(37)_ = -0.29, p = 0.773, anecdotal evidence against the effect  (BF_10_/BF_01_ = 0.34/2.97). |
| **Execution time** | **Post** | t_(13)_ = 2.42, p = 0.031,  B_0_ = 10%, d_z_ = 0.62. | t_(24)_ = 4.75, p < 0.001,  B_0_ = 14%, d_z_ = 0.93. | t_(38)_ = 0.16, p = 0.876,  substantial evidence against the effect  (BF_10_/BF_01_ = 0.31/3.18). | t_(37)_ = -0.07, p = 0.943, substantial evidence against the effect  (BF_10_/BF_01_ = 0.32/3.16). |
|  | **Ret.** | t_(13)_ = 2.40, p = 0.032,  B_0_ = 9%, d_z_ = 0.62. | t_(24)_ = 3.99, p = 0.001,  B_0_ = 10%, d_z_ = 0.78. | t_(38)_ = -0.47, p = 0.64,  anecdotal evidence against the effect  (BF_10_/BF_01_ = 0.35/2.84). | t_(37)_ = -0.42, p = 0.678, anecdotal evidence against the effect  (BF_10_/BF_01_ = 0.35/2.82). |
| **Error rate** | **Post** | t_(13)_ = 1.90, p = 0.079,  B_0_ = 6%, d_z_ = 0.49. | t_(24)_ = 2.89, p = 0.008,  B_0_ = 9%, d_z_ = 0.57. | t_(38)_ = 0.69, p = 0.497,  anecdotal evidence against the effect  (BF_10_/BF_01_ = 0.38/2.63). | t_(37)_ = 0.43, p = 0.667,  anecdotal evidence against the effect  (BF_10_/BF_01_ = 0.36/2.82). |
|  | **Ret.** | t_(13)_ = 2.13, p = 0.053,  B_0_ = 8%, d_z_ = 0.55. | t_(24)_ = 1.45, p = 0.161,  B_0_ = 4%, d_z_ = 0.28. | t_(38)_ = -0.72, p = 0.476,  anecdotal evidence against the effect  (BF_10_/BF_01_ = 0.38/2.61). | t_(37)_ = -1.00, p = 0.322, anecdotal evidence against the effect  (BF_10_/BF_01_ = 0.46/2.20). |
| Shaded fields highlight non-significant effects. | | | | | |


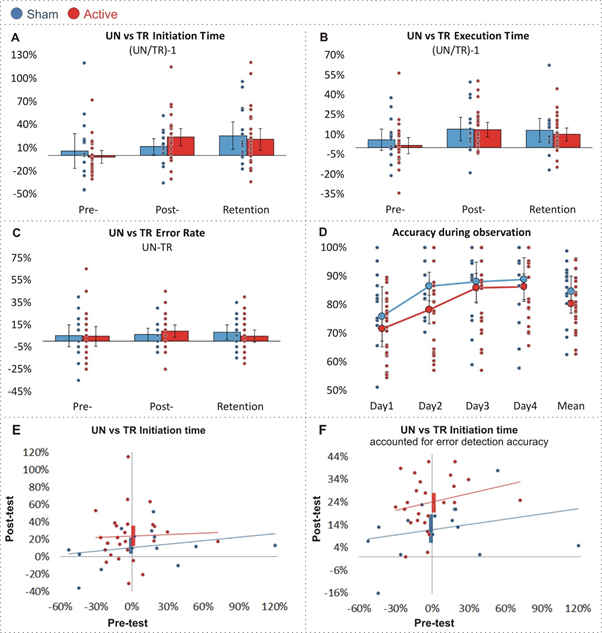


Figure 1. Performance results. Pre-, post-, and retention-test difference in initiation time (A), execution time (B), and error rate (C) between trained (TR) and untrained (UN) sequences for sham (blue) and active (red) stimulation groups. D. Error detection accuracy during observational practice sessions. A-D. Bars and large dots: group averages; small dots: individual participant values; error bars: 95% CI. E. Regression lines of pre-test (predictor) and the post-test difference between trained and untrained sequence initiation times for sham (blue) and active (red) stimulation groups. Intercepts of the regression lines represent the predicted post-test difference if the pre-test difference is zero. Vertical bars represent 96% CIs of intercepts F. Same as E, but post-test difference corrected for error detection accuracy during training sessions.

## Supplementary Materials 2: Raw performance measures


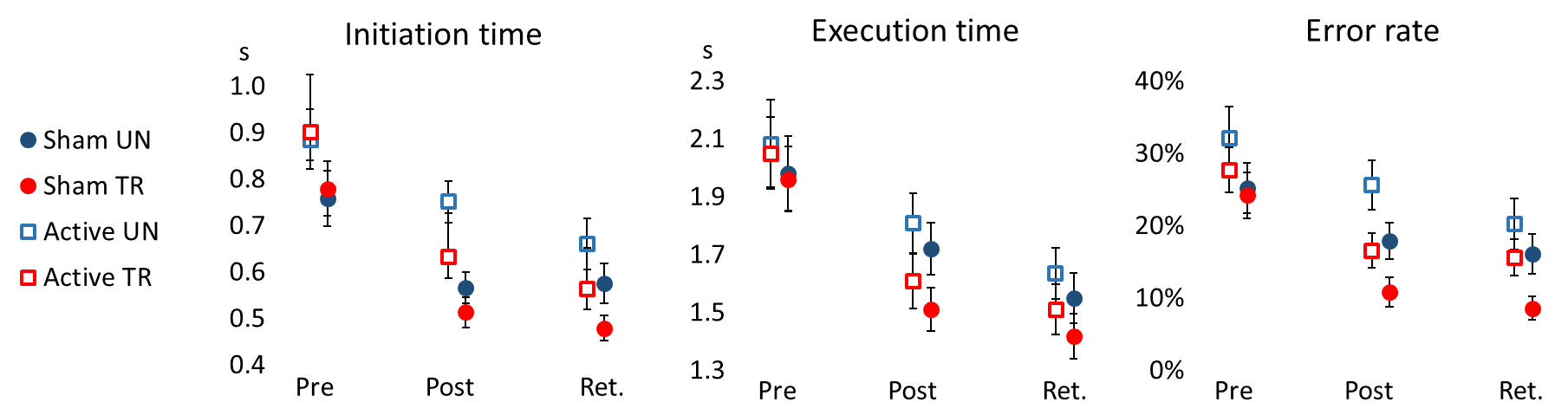


The figure above illustrates the mean and standard deviation values for participants’ initiation time, execution time, and error rate (three different panels), split into Pre, Post and Retention tests (along x-axes). These data are further split into stimulation groups (active vs. sham; filled circles vs. open squares) and the training status of the sequences (blue for untrained and red for trained). Error bars represent standard error of the means.

## Supplementary Materials 3: Group differences in performance generalisation to the untrained sequences

The table below shows independent sample t-test significance comparing sham and active stimulation group differences in performance generalisation to the untrained sequences.

| **p-value** | |
| --- | --- |
| **Pre vs . Post difference of untrained sequence** | |
| Initiation time | 0.107 |
| Execution time | 0.847 |
| Error rate | 0.901 |
| **Pre vs. Retention difference of untrained sequence** | |
| Initiation time | 0.515 |
| Execution time | 0.936 |
| Error rate | 0.674 |
| **Accounted for the accuracy during training sessions** | |
| **Pre vs. Post difference of untrained sequence** | |
| Initiation time | 0.045 |
| Execution time | 0.784 |
| Error rate | 0.982 |
| **Pre vs. Retentions difference of untrained sequence** | |
| Initiation time | 0.661 |
| Execution time | 0.909 |
| Error rate | 0.596 |

## Supplementary Materials 4: tDCS effects on sequence-specific learning using ANCOVA

**Dependent variable**: Post-training (separate for post-test and retention-test) trained/untrained sequence performance difference, UN/TR-1 for the IT and ET and UN-TR for Err.

**Covariate**: Pre-training trained/untrained sequence performance difference, UN/TR-1 for the IT and ET and UN-TR for Err.

**Within-subject factor:** stimulation (active/sham).

Significance reported one-tailed

**Post-test**

**Initiation time**

| **Tests of Between-Subjects Effects** | | | | | |
| --- | --- | --- | --- | --- | --- |
| Dependent Variable: ITPostDiff | | | | | |
| Source | Type III Sum of Squares | df | Mean Square | F | Sig. |
| Corrected Model | .186^a^ | 2 | .093 | 1.293 | .142 |
| Intercept | 1.669 | 1 | 1.669 | 23.250 | .000 |
| ITPreDiff | .037 | 1 | .037 | .510 | .240 |
| **stimulation** | **.161** | **1** | **.161** | **2.238** | **.072** |
| Error | 3.374 | 47 | .072 |  |  |
| Total | 5.286 | 50 |  |  |  |
| Corrected Total | 3.560 | 49 |  |  |  |
| a. R Squared = .052 (Adjusted R Squared = .012) | | | | | |

**Execution time**

| **Tests of Between-Subjects Effects** | | | | | |
| --- | --- | --- | --- | --- | --- |
| Dependent Variable: ETPostDiff | | | | | |
| Source | Type III Sum of Squares | df | Mean Square | F | Sig. |
| Corrected Model | .043^a^ | 2 | .021 | 1.009 | .187 |
| Intercept | 1.002 | 1 | 1.002 | 47.007 | .000 |
| ETPreDiff | .041 | 1 | .041 | 1.915 | .087 |
| **stimulation** | **.003** | **1** | **.003** | **.139** | **.355** |
| Error | 1.001 | 47 | .021 |  |  |
| Total | 2.077 | 50 |  |  |  |
| Corrected Total | 1.044 | 49 |  |  |  |
| a. R Squared = .041 (Adjusted R Squared = .000) | | | | | |

**Error rate**

| **Tests of Between-Subjects Effects** | | | | | |
| --- | --- | --- | --- | --- | --- |
| Dependent Variable: ErrPostDiff | | | | | |
| Source | Type III Sum of Squares | df | Mean Square | F | Sig. |
| Corrected Model | .005^a^ | 2 | .003 | .130 | .439 |
| Intercept | .315 | 1 | .315 | 15.292 | .000 |
| ErrPreDiff | .001 | 1 | .001 | .029 | .433 |
| **stimulation** | **.004** | **1** | **.004** | **.217** | **.322** |
| Error | .969 | 47 | .021 |  |  |
| Total | 1.303 | 50 |  |  |  |
| Corrected Total | .974 | 49 |  |  |  |
| a. R Squared = .006 (Adjusted R Squared = -.037) | | | | | |

**Retention-test**

**Initiation time**

| **Tests of Between-Subjects Effects** | | | | | |
| --- | --- | --- | --- | --- | --- |
| Dependent Variable: ITRetDiff | | | | | |
| Source | Type III Sum of Squares | df | Mean Square | F | Sig. |
| Corrected Model | .046^a^ | 2 | .023 | .198 | .411 |
| Intercept | 2.197 | 1 | 2.197 | 18.750 | .000 |
| ITPreDiff | .046 | 1 | .046 | .395 | .267 |
| **stimulation** | **.000** | **1** | **.000** | **.002** | **.480** |
| Error | 5.506 | 47 | .117 |  |  |
| Total | 7.771 | 50 |  |  |  |
| Corrected Total | 5.553 | 49 |  |  |  |
| a. R Squared = .008 (Adjusted R Squared = -.034) | | | | | |

**Execution time**

| **Tests of Between-Subjects Effects** | | | | | |
| --- | --- | --- | --- | --- | --- |
| Dependent Variable: ETRetDiff | | | | | |
| Source | Type III Sum of Squares | df | Mean Square | F | Sig. |
| Corrected Model | .062^a^ | 2 | .031 | 1.755 | .092 |
| Intercept | .476 | 1 | .476 | 27.028 | .000 |
| ETPreDiff | .062 | 1 | .062 | 3.511 | .034 |
| **stimulation** | **6.958E-5** | **1** | **6.958E-5** | **.004** | **.475** |
| Error | .827 | 47 | .018 |  |  |
| Total | 1.392 | 50 |  |  |  |
| Corrected Total | .889 | 49 |  |  |  |
| a. R Squared = .070 (Adjusted R Squared = .030) | | | | | |

**Error rate**

| **Tests of Between-Subjects Effects** | | | | | |
| --- | --- | --- | --- | --- | --- |
| Dependent Variable: ErrRetDiff | | | | | |
| Source | Type III Sum of Squares | df | Mean Square | F | Sig. |
| Corrected Model | .023^a^ | 2 | .011 | .615 | .273 |
| Intercept | .168 | 1 | .168 | 9.105 | .002 |
| ErrPreDiff | .012 | 1 | .012 | .668 | .209 |
| **stimulation** | **.012** | **1** | **.012** | **.662** | **.210** |
| Error | .867 | 47 | .018 |  |  |
| Total | 1.070 | 50 |  |  |  |
| Corrected Total | .890 | 49 |  |  |  |
| a. R Squared = .026 (Adjusted R Squared = -.016) | | | | | |
